# Effect of High-Intensity Training in Normobaric Hypoxia on Thoroughbred Skeletal Muscle

**DOI:** 10.1155/2016/1535367

**Published:** 2016-09-19

**Authors:** Hiroshi Nagahisa, Kazutaka Mukai, Hajime Ohmura, Toshiyuki Takahashi, Hirofumi Miyata

**Affiliations:** ^1^Biological Sciences, Graduate School of Medicine, Yamaguchi University, Yoshida 1677-1, Yamaguchi 753-8515, Japan; ^2^Equine Research Institute, Japan Racing Association, 1400-4 Shiba, Shimotsuke, Tochigi 329-0412, Japan

## Abstract

Hypoxic training is believed to increase endurance capacity in association with hypoxia inducible factor-1*α* (HIF-1*α*), a modulator of vascular endothelial growth factor-A (VEGF-A), and to influence activation of satellite cells (SCs). However, the effect of hypoxic training on SC activation and its relation to angiogenesis has not been thoroughly investigated. Eight Thoroughbred horses were subjected to normoxic (F_IO2_ = 21%) or hypoxic (F_IO2_ = 15%) training for 3 days/week (100%  ${\dot{V}O}_{2}max$) for 4 weeks. Incremental exercise tests (IET) were conducted on a treadmill under normoxia and the maximal oxygen consumption (${\dot{V}O}_{2}max$) and running distance were measured before and after each training session. Muscle biopsy samples were obtained from the gluteus medius muscle at 6 scheduled times before, during, and one week after IET for immunohistochemical analysis and real-time RT-PCR analysis. Running distance and ${\dot{V}O}_{2}max$, measured during IET, increased significantly after hypoxic training compared with normoxic training. Capillary density and mRNA expression related to SC activation (e.g., myogenin and hepatocyte growth factor) and angiogenesis (VEGF-A) increased only after hypoxic training. These results suggest that increases in mRNA expression after training enhance and prolong SC activation and angiogenesis and that nitric oxide plays an important role in these hypoxia-induced training effects.

## 1. Introduction

Hypoxic training is commonly used to increase endurance capacity in athletes [1–3]. By contrast, long-term (4–8 weeks) or severe exposure (4000–5000 m altitude) to hypoxia results in deterioration of skeletal muscle tissue and impairment of vascular hemodynamics [1, 2, 4]. As a method of utilizing hypoxic training without this negative effect, the “live-low, train-high” theory has been proposed [1]. This type of training is intended to gain synergistic training effects through intensive metabolic stress combined with exercise and hypoxia. A previous study, based on this theory, demonstrated improvements in maximal oxygen consumption (${\dot{V}O}_{2}max$) and increased capillary density related to the upregulation of vascular endothelial growth factor-A (VEGF-A) expression [1].

Many studies at the cellular level demonstrated that hypoxia inducible factor-1*α* (HIF-1*α*) had an important role in structural and metabolic adaptation to hypoxia and that hypoxia has been mainly investigated in association with HIF-1*α*, which stabilizes under hypoxia and leads to angiogenesis via upregulation of VEGF-A [5, 6]. Furthermore, satellite cells (SCs) are localized under the basal lamina and are myogenic stem cells related to muscle repair and angiogenesis [7–9]. SCs are close to capillaries and secrete growth factors involved in both angiogenesis and myogenesis [7]. SCs are normally quiescent but are activated and proliferate in response to internal and external stimuli. It was demonstrated that, in a hypoxic environment, activation and proliferation of SCs are facilitated regardless of inhibition of their differentiation [10–12]. Furthermore, exercise training increased the number of SCs and enhanced the reactivity of SC-related genes [13]. Based on these findings, angiogenesis in skeletal muscle induced by hypoxic training is, at least partly, associated with activation of SCs. However, there is little information concerning the relationship between SCs and angiogenesis in hypoxia training.

Our final goal is to disclose the effect of hypoxia training on mammals including the human. To analyze muscle properties with multiple biopsy sampling, horses are a superior model due to the large muscle volume. Up to now, there are few studies about hypoxia-training effect on horse muscles. In this study, we examined whether chronic exercise in normobaric hypoxia could enhance the activation of SCs, angiogenesis, and mitochondrial biogenesis in equine skeletal muscle.

## 2. Materials and Methods

### 2.1. Animals and Training Protocol

All procedures were approved by the Animal Welfare and Ethics Committee of the Japan Racing Association Equine Research Institute and followed the American Physiological Society's Animal Care Guidelines.

Eight trained Thoroughbred horses (5 geldings and 3 mares, 6.5 ± 1.7 years old, 502 ± 14 kg) were used in this study. The horses were trained to run on a treadmill (Sato I, Sato AB, Uppsala, Sweden) while wearing an open-flow mask [14] and were kept in 2 ha pastures for approximately 6 hours/day every day for at least 4 months before treadmill experiments began.

**Figure 1:**
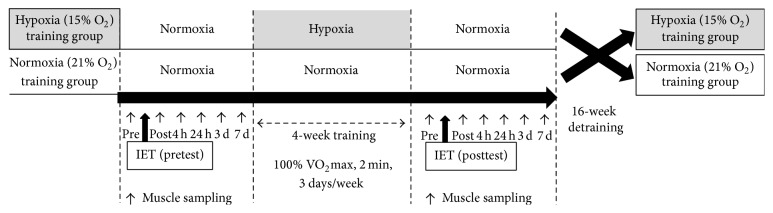
Schematic figure of the experimental schedule. The training protocol adopted a randomized crossover design, which was separated by a 16-week detraining period. Eight horses were assigned randomly into normoxic training (*n* = 4, F_IO2_ = 21%) and hypoxic training (*n* = 4, F_IO2_ = 15% O_2_) groups. Incremental exercise tests (IET) were carried out before (pretest) and after (posttest) training. In each IET, horses were subjected to biopsy sampling of the gluteus medius six times, indicated by “↑” (before (pre) and immediately (post) and 4 hours (4 h), 24 hours (24 h), 3 days (3 d), and 7 days (7 d) after IET).

The training protocol adopted a randomized crossover design that was separated by a 16-week detraining period (Figure 1). Horses were divided randomly into normoxic training (*n* = 4, F_IO2_ = 21%) and hypoxic training (*n* = 4, F_IO2_ = 15%  O_2_) groups. They were trained 3 days/week on a treadmill inclined at 6% and were pastured in 2-hectare pastures for approximately 6 hours/day on the other 4 days for 4 weeks. The training session consisted of warm-up (1.7 m/s for 1 min and 4 m/s for 2 min), cantering (7 m/s for 1 min), at 100%$\;\;{\dot{V}O}_{2}max$ (11.7 ± 0.2 m/s) measured under normoxia (approximately 2 min), and cooldown (1.7 m/s for 3 min) in both groups.

### 2.2. Incremental Exercise Test (IET)

Before and after each training period, all horses were subjected to an incremental exercise test (IET) under normoxia. The first IET (pretest) was used as the control (normoxic training group pretest: Nor Con; hypoxic training group pretest: Hypo Con) and the second IET (posttest) was performed after completing the training period (normoxic training group posttest: Nor Tr; hypoxic training group posttest: Hypo Tr).

Horses ran on a treadmill inclined at 6% to determine the speed at which each horse would reach exhaustion. To assess the state of exhaustion of each horse, heart rate (S810, Polar, Kempele, Finland) and running attitude during the treadmill exercise were observed by horse trainers. After walking at 1.7 m/s and trotting at 3.5 m/s to warm up, horses ran at 6 m/s for 2 minutes. Then, the speed was increased by 2 m/s every two minutes, until each horse reached exhaustion that the horse could not maintain its position at the front of the treadmill with humane encouragement. For each speed, the horse ran on the treadmill for 90 s to allow the O_2_ transport system to come to steady state, an then ${\dot{V}O}_{2}$ was calculated for the final 30 s of each step. We measured the distance covered and ${\dot{V}O}_{2}$ during IET.

### 2.3. Muscle Sampling and Immunohistochemical Analysis

In each IET, muscle samples (~50 mg wet weight) were obtained from the same area (concentrically 2 cm away from the first sampling point) at the midsection of the gluteus medius muscle [15] and from the same depth (5 cm below the skin surface) by needle biopsy under local anesthesia (lidocaine, Fujisawa Pharmaceutical Co., Osaka, Japan) before exercise (pre) and immediately (post) and 4 hours (4 h), 24 hours (24 h), 3 days (3 d), and 7 days (7 d) after exercise. All muscle samples were frozen by liquid nitrogen and stored at −80°C until analyzed.

The fiber type population was determined as previously described [16]. Serial 7 *μ*m cross sections of the muscle were obtained on a cryostat (CM510, Leica, Nussloch, Germany) at −20°C. The sections were warmed to room temperature (RT) and then preincubated in 1% normal goat serum (Millipore-Chemicon, Billerica, MA, USA) in 0.1 M phosphate buffered saline (PBS, pH 7.6) at RT for 10 min. The primary monoclonal antibody was then applied, either (1) fast myosin (Sigma, St. Louis, USA; 1 : 4,000), which specifically reacts with myosin heavy chain-IIa (MHC-IIa) and MHC-IIx, or (2) SC-71 (Developmental Studies Hybridoma Bank, Iowa, USA; 1 : 1,000), which specifically reacts with MHC-IIa. The sections were incubated in these primary antibodies overnight at RT and incubated with a secondary antibody (goat anti-mouse IgG) conjugated with horseradish peroxidase (HRP, Bio-Rad, Hercules, CA, USA; 1 : 1,000) at RT for 3 hours. Diaminobenzidine tetrahydrochloride (Bio-Rad) was used as a chromogen to localize HRP. Images of the stained muscle fibers were recorded with a photomicroscopic (E600, Nikon, Tokyo, Japan) image-processing system (DS-U1, Nikon). The fibers were classified as type I, IIa, or IIx fibers based on their immunohistochemical staining properties, and the population percentage and cross-sectional area of each muscle fiber type were calculated from at least 300 muscle fibers.

### 2.4. Satellite Cell and Capillary Identification

**Figure 2:**
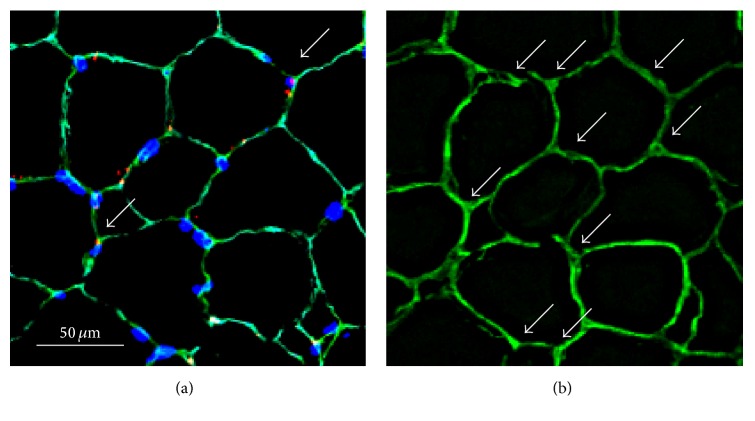
Typical photomicrographs of serial transverse sections of the gluteus medius muscle. Thicknesses of sections are 7 and 50 *μ*m in panels (a) and (b), respectively. (a) Triple-immunofluorescent stained for laminin (green), Pax7 (red), and nuclei (blue). The white arrow in (a) indicates a satellite cell (Pax7^+^ nuclei). (b) Single-immunofluorescent stained for laminin. White arrows in (b) indicate capillaries.

The procedures for the determination of satellite cell number and capillary density were identical to those described previously [17]. Another serial 7 *μ*m cross section and one 50 *μ*m cross section of the muscle were obtained on a cryostat at −20°C. The 7 *μ*m section was fixed in 4% paraformaldehyde in 0.1 M PBS at RT for 10 min. These sections were preincubated in blocking solution containing 10% normal goat serum and 0.1% and 2% bovine serum albumin in PBS at RT for 30 min. Each section was incubated for 1 hour at RT in the primary antibodies, mouse anti-paired box protein-7 (Pax7, Developmental Studies Hybridoma Bank; 1 : 1,000) and rabbit anti-laminin (Sigma, 1 : 1,000) diluted in 2% bovine serum albumin/PBS. The sections were incubated in appropriate secondary antibodies: Cy3-conjugated AffiniPure goat anti-mouse IgG (Jackson ImmunoResearch, West Grove, USA; 1 : 1,000) for Pax7 and Alexa Fluor 488 goat anti-rabbit IgG (Molecular Probes, Breda, Netherlands; 1 : 1,000) for laminin. After incubation, the sections were stained with 4,6-diamidino-2-phenylindole (DAPI) diluted in PBS at RT for 5 min. Images for anti-Pax7, anti-laminin, and DAPI were merged with image-processing software (Adobe Photoshop Elements 12, Adobe, San Jose, CA, USA) and used for quantification of SCs (Figure 2(a)). SCs were identified as being positive for both DAPI and Pax7 at the periphery of each fiber beneath the basal lamina. The numbers of SCs/fibers were calculated for the 3 fiber types separately.

The 50 *μ*m section was immunohistochemically processed with the primary antibody (anti-laminin) and the following secondary antibody (Alexa Fluor 488 goat anti-rabbit IgG) as mentioned above. At least 4 areas in a muscle sample were imaged using a laser-scanning confocal system (C1, Nikon). At least 15 images of optical sections at 2.0 *μ*m steps were obtained from an area, and two-dimensional (2D) stacked images for each area were reconstructed (Figure 2(b)). The capillaries surrounding clear basal lamina were counted and presented as capillary density (the number of capillaries per 1 mm^2^).

### 2.5. RNA Isolation and Real-Time RT-PCR

Total RNA was extracted from each muscle sample with TRIzol reagent (Molecular Probes, Breda, Netherlands). The purity and quantity of total RNA were determined by measuring the absorbance of aliquots at 260 and 280 nm. Total RNA was then treated for 30 min at 37°C with TURBO DNase (Ambion, Austin, USA) to remove genomic DNA from samples. DNase-treated RNA (0.5 *μ*g) was used to synthesize first-strand cDNA with Exscript*™* RT reagent kit (Takara, Tokyo, Japan). Thereafter, the cDNA products were analyzed by real-time PCR using the SYBR Green PCR Master Mix protocol in StepOne*™* Real-Time PCR System (Applied Biosystems Japan, Tokyo, Japan).

The amplification program included an initial denaturation step at 95°C for 10 min, 40 cycles of denaturation at 95°C for 30 sec, and annealing/extension at 58°C for 1 min. The amount of glyceraldehyde-3-phosphate dehydrogenase (GAPDH) mRNA was estimated as an internal control. Each mRNA was normalized to GAPDH by subtracting the cycle threshold (Ct) value of GAPDH from the Ct value of the gene target [ΔCt (target)]. The relative expression of the target gene was calculated as the relative quantification value for the Pre value. Following the relative expression, dissociation-curve analysis detected no nonspecific amplification in cDNA samples.

The sequences of the specific primers used in this study are presented in Table 1. Each PCR primer was designed by Primer Express® software (Applied Biosystems Japan), and the oligonucleotides were purchased from FASMAC (FASMAC, Kanagawa, Japan).

### 2.6. Statistics

All data are presented as the mean ± SEM. A paired *t*-test was used to compare differences between control and training in all data and between normoxic and hypoxic conditions in mRNA expression data. In both cases, the statistical significance was set at *p* < 0.05.

## 3. Results

### 3.1. Body Weight and Exercise Ability

**Figure 3:**
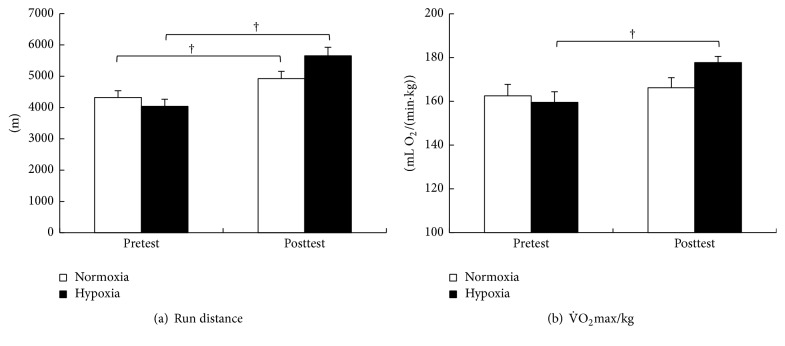
Changes in run distance (a) and maximal oxygen consumption (${\dot{V}O}_{2}max$) (b) in the incremental exercise test under normoxia for the normoxic training group (white bar) and the hypoxic training group (black bar). ^†^Significant difference versus pretest (*p* < 0.05). Values are mean ± SEM.

Body weight was significantly reduced in both groups after training (Nor: from 498 ± 6 to 489 ± 8 kg; *p* = 0.034; Hypo: from 499 ± 6 to 483 ± 7 kg; *p* = 0.006). As shown in Figure 3(a), although running distance in IET increased after training under both oxygen conditions, a larger increase (40.1%) was observed in Hypo Tr (from 4036 ± 225 to 5653 ± 266 m; *p* = 0.0001) than the value (14.0%) in Nor (from 4317 ± 217 to 4921 ± 232 m; *p* = 0.0006). As shown in Figure 3(b), although ${\dot{V}O}_{2}max$ in IET increased after training under both oxygen conditions, significant increases (11.4%) were observed only in Hypo Tr (from 159.5 ± 4.9 to 177.7 ± 2.8 mL/min/kg; *p* = 0.011), but not in Nor (from 162.5 ± 5.3 to 166.2 ± 4.6 mL/min/kg).

### 3.2. Muscle Fiber Properties

**Figure 4:**
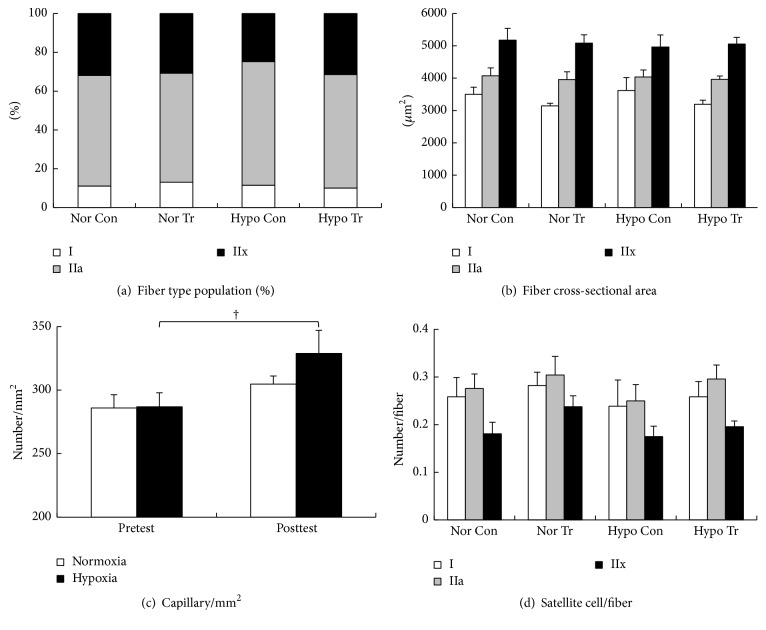
Changes in fiber type population (a), fiber cross-sectional area (b), capillary density (c), and number of satellite cells (d) in pretest of control (Nor Con: normoxic control; Hypo Con: hypoxic control) and posttest (Nor Tr: normoxic training; Hypo Tr: hypoxic training) in normoxic or hypoxic training group. Measurements were performed on muscle samples obtained before the incremental exercise test (pre). White, grey, and black bars in (a), (b), and (d) represent fiber types I, IIa, and IIx, respectively. White and black bars in (c) represent normoxia and hypoxia, respectively. ^†^Significant difference versus pretest (*p* < 0.05). Values are mean ± SEM.

Regardless of oxygen concentration and training, as shown in Figure 4(a), fiber type populations at Pre were similar for all conditions (type I: 11.0–13.1%; type IIa: 57.0–63.8%; type IIx: 24.7–31.4%). Likewise, as shown in Figure 4(b), cross-sectional areas of each fiber type were similar for all conditions (type I: 3145–3621 *μ*m^2^; type IIa: 3956–4072 *μ*m^2^; type IIx: 4967–5176 *μ*m^2^). As shown in Figure 4(c), although capillary density increased after training under both oxygen conditions, significant increases were found in only Hypo Tr (Nor: from 286 ± 11 to 304 ± 6/mm^2^; Hypo: from 287 ± 11 to 329 ± 18/mm^2^; *p* = 0.045). As shown in Figure 4(d), SC number/fiber slightly increased after training in all fiber types in Nor Tr (type I: from 0.26 ± 0.04 to 0.28 ± 0.03; type IIa: from 0.28 ± 0.03 to 0.30 ± 0.04; type IIx: from 0.18 ± 0.02 to 0.24 ± 0.02) and Hypo Tr (type I: from 0.24 ± 0.06 to 0.26 ± 0.03/fiber; type IIa: from 0.25 ± 0.03 to 0.30 ± 0.03/fiber; type IIx: from 0.17 ± 0.02 to 0.2 ± 0.01/fiber).

### 3.3. Expression of mRNA

**Figure 5:**
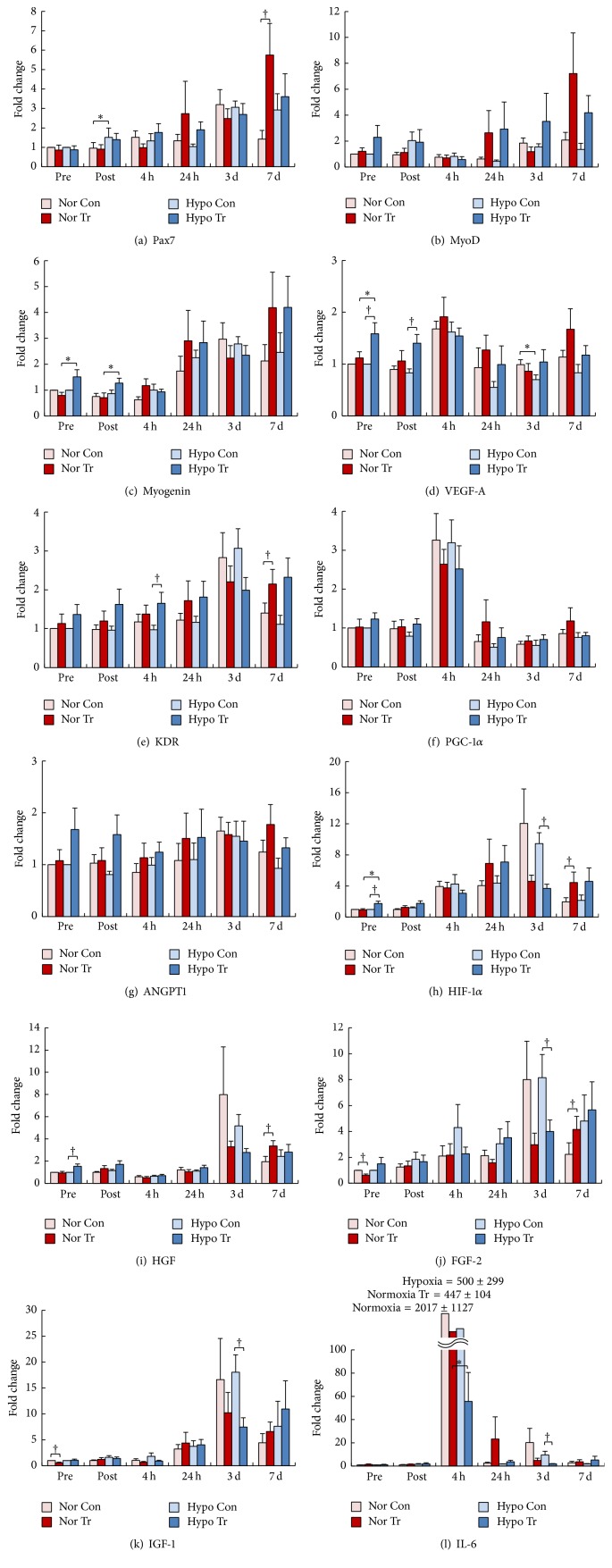
(a–f) Time-course changes in mRNA expression of Pax7 (a), MyoD (b), myogenin (c), VEGF-A (d), KDR (e), and PGC-1*α* (f) in pretest of control (Nor Con: normoxic control, light red; Hypo Con: hypoxic control, light blue) and posttest (Nor Tr: normoxic training, red; Hypo Tr: hypoxic training, blue) after normoxic or hypoxic training. Measurements were performed before (pre) and immediately (post) and 4 hours (4 h), 24 hours (24 h), 3 days (3 d), and 7 days (7 d) after the incremental exercise test. Values of mRNA expression were calculated as x-fold change from pretest of each control. ^†^Significant difference versus pretest (*p* < 0.05). Values are mean ± SEM. ^*∗*^Significant difference versus normoxia training group (*p* < 0.05). (g–l) Time-course changes in mRNA expression of ANGPT1 (g), HIF-1*α* (h), HGF (i), FGF-2 (j), IGF-1 (k), and IL-6 (l) in pretest of control (Nor Con: normoxic control, light red; Hypo Con: hypoxic control, light blue) and posttest (Nor Tr: normoxic training, red; Hypo Tr: hypoxic training, blue) after normoxic or hypoxic training. Measurements were performed before (pre) and immediately (post) and 4 hours (4 h), 24 hours (24 h), 3 days (3 d), and 7 days (7 d) after the incremental exercise test. Values of mRNA expression were calculated as x-fold change from pretest of each control. ^†^Significant difference versus pretest (*p* < 0.05). Values are mean ± SEM. ^*∗*^Significant difference versus normoxic training group (*p* < 0.05).

Relative changes in mRNA expression from Pre values of each control are presented in Figure 5. Pax7 mRNA expression was similar for all Pre conditions and then increased gradually to 7 d after IET. Although there were no significant differences between each condition, myogenic determination factor (MyoD) mRNA expression increased 2.3 times in Pre in Hypo Tr. Time-course changes in MyoD expression, in addition to a decrease 4 h after training in all groups, tended to increase 24 h and 7 d after both training regimens. Compared to Nor Tr, myogenin mRNA increased significantly in Pre (*p* = 0.023) and Post (*p* = 0.002) in Hypo Tr. Although no significant differences were detected, expression of myogenin mRNA showed a similar tendency in Pax7 and MyoD mRNA, with increases at 24 h and 7 d after training.

VEGF-A mRNA expression in Hypo Tr significantly increased in Pre (*p* = 0.029) and Post (*p* = 0.020) compared to Hypo Con. In all conditions, VEGF-A mRNA expression tended to increase at 4 h and increased 24 h and 7 d after training. Compared with controls, mRNA expression of VEGF receptor-2 (KDR) significantly increased at 4 h (*p* = 0.031) in Hypo Tr and at 7 d (*p* = 0.016) in Nor Tr. Peroxisome proliferator-activated receptor coactivator-1*α* (PGC-1*α*) mRNA expression tended to increase 4 h after training, as demonstrated by VEGF-A mRNA expression. PGC-1*α* mRNA expression tended to decrease in the late stage (24 h~3 d). Although significant changes in angiopoietin-1 (ANGPT1) mRNA expression were not observed between each condition, mRNA expression tended to be elevated in Pre and Post in Hypo Tr. In Hypo Tr, HIF-1*α* mRNA expression in Pre increased significantly as compared to Hypo Con (*p* = 0.042) and Nor Tr (*p* = 0.041) and decreased significantly 3 d (*p* = 0.007) after training compared to Hypo Con. Furthermore, HIF-1*α* mRNA expression in Nor Tr significantly increased at 7 d (*p* = 0.040) compared to Nor Con. Under all oxygen conditions, time-course changes in HIF-1*α* expression after training tended to increase in the late stage (24 h~7 d).

The time-course changes in hepatocyte growth factor (HGF) and HIF-1*α* mRNA expression were similar. Compared to controls, HGF mRNA expression significantly increased in Pre (*p* = 0.037) in Hypo Tr and at 7 d (*p* = 0.023) in Nor Tr. In addition to these changes, mRNA expression of HGF tended to decrease, as shown by MyoD mRNA expression at 4 h. Relative changes in mRNA expression were similar in fibroblast growth factor-2 (FGF-2) and insulin-like growth factor-1 (IGF-1) and HGF and HIF-1*α*. In Hypo Tr, FGF-2 mRNA expression significantly decreased compared to the control at 3 d (*p* = 0.013). Additionally, compared to the control, FGF-2 mRNA expression in Nor Tr decreased significantly in Pre (*p* = 0.007) and increased at 7 d (*p* = 0.033). Significant decreases in mRNA expression of IGF-1 were found in Hypo at 3 d (*p* = 0.026) and in Nor in Pre (*p* = 0.010) as compared to each control; these changes were very similar to those of FGF-2. Considerable increases of mRNA expression at 3 d were observed in SC activators, that is, HGF, FGF-2, IGF-1, and HIF-1*α*. In all conditions, mRNA expression of interleukin-6 (IL-6) greatly increased at 4 h. However, the time-course changes in IL-6 mRNA in Hypo Tr decreased significantly at 4 h (*p* = 0.006) and 3 d (*p* = 0.040), compared to Nor Tr and the control, respectively.

## 4. Discussion

### 4.1. Improvements of Endurance Ability by Hypoxic Training

In this study, significant improvements were observed in running distance and ${\dot{V}O}_{2}max$ during IET and capillary density after hypoxic training over normoxic training. Upregulation of angiogenesis factors such as VEGF-A and ANGPT1 was responsible for the increase in capillary density. Our hypoxic training significantly enhanced VEGF-A mRNA expression before and after IET. A previous study [18] demonstrated that, in trained rats with ligated femoral artery, the number of capillary contacts per muscle fiber increased at 12 days after training, while VEGF-A mRNA increased until 8 days after training and returned to basal level. Additionally, increased VEGF-A protein without rise of the mRNA was shown in human muscles at 5 weeks after the training with blood flow restriction [19]. These results suggest that immediate upregulation of transcription of VEGF-A mRNA after training is associated with increased translation and that the increase of VEGF-A protein is essential for angiogenesis. Furthermore, we reported that time-course changes in VEGF-A mRNA expression were related to PGC-1*α* mRNA expression when exercise tests were performed under acute exposure to normobaric hypoxia in a previous study [17]. Likewise, this study also demonstrated that changes in VEGF-A and PGC-1*α* mRNA expression are similar. However, although PGC-1*α* mRNA expression increased at 4 h for Hypo Tr, further upregulation of VEGF-A mRNA was not observed. This is in agreement with our previous results that resting and exercise-induced upregulation of VEGF-A mRNA and protein levels had been attenuated after training [20]. This attenuation of exercise-induced upregulation of VEGF-A is considered to be a training adaptation.

It has been reported that hypoxic training enhances NO (nitric oxide) production (measured by nitrite/nitrate) and expression of NO synthase (NOS) [21, 22]. Although we did not determine certain factors related to NO, it has been shown to have anti-inflammatory effects and to decrease IL-6 mRNA expression [23]. In [23], administration of NOS inhibitor into pharmacologically induced damaged muscle significantly increased IL-6 mRNA expression 4 and 7 days after injury. In our study also, IL-6 mRNA expression significantly decreased at 4 h and 3 d as a result of hypoxic training, indicating that hypoxic training results in greater generation of NO than normoxic training.

HIF-1*α* is an upregulation factor for VEGF-A. Under normoxia, HIF-1*α* is hydroxylated by prolyl hydroxylase (PHD), which requires an oxygen ligand, and is eventually degraded by proteasomes [6, 24]. NO prevents degradation of HIF-1*α* under normoxia, in association mainly with PHD [24]. This stabilization of HIF-1*α* via NO is reversed under hypoxia, and NO prevents oxygen consumption by mitochondrial complex IV, thus allowing oxygen to be supplied to PHD [24]. Thus, HIF-1*α* protein is regulated by oxygen concentration or NO. In this study, time-course changes in HIF-1*α* and HGF, IGF-1, and FGF-2 mRNA expression were similar. HGF is involved in the upregulation of HIF-1*α* mRNA [25]; and IGFs [9] and FGF-2 [26] are associated with the regulation of HIF-1*α*. Probably, due to the regulation of the protein level, changes in HIF-1*α* mRNA which were observed in the present study were not similar to changes in VEGF-A mRNA. However, time-course changes in mRNA expression were similar for HIF-1*α* and its receptor, KDR. These results suggest the possibility that mRNA expression of both factors is regulated simultaneously.

### 4.2. Satellite Cell Activation by Hypoxic Training

SCs lie adjacent to capillaries and are activated by endothelial cell-derived growth factors (HGF, VEGF, IGF-1, and FGF-2) that promote SC proliferation [7]. During the transition from quiescence to activation and differentiation, SCs release VEGF, ANGPT1, FGF-2, and HGF, which have a proangiogenic effect on endothelial cells [7–9]. This effect on endothelial cells is most intense during SC differentiation, while myogenin is being expressed, in parallel to an increase in factors, such as VEGF and ANGPT1 [7, 8]. Additionally, a previous study hypothesized that SCs act as vehicles for muscle-regulated angiogenesis and that SC activation is needed for muscle repair and angiogenesis [9]. Under hypoxia, SC is activated mainly by HIF-1*α*, while its differentiation is blocked [10–12]. Transient hypoxic activation of SCs, due to the stabilization of HIF-1*α*, and the subsequent degradation of HIF-1*α*, which blocks SC differentiation, were promoted by a gradual increase in oxygen [10]. This demonstrates that* in vivo* muscle repair was initiated more quickly by transient hypoxia than by chronic hypoxia and normoxia [10]. As mentioned above, NO is related to the stabilization and degradation of HIF-1*α*. NO also regulates the release of HGF, which activates SCs [9] and upregulates HIF-1*α* mRNA [25]. We speculate that if NOS and the plasma nitrite/nitrate level are increased by hypoxic training, greater SC activation and proliferation will be observed in the Hypo Tr group during the IET under normoxia. As the results of the present study show, SC number/fiber in all muscle fiber types tended to increase after training, and a significant increase in capillary density was found only in Hypo Tr.

In injured muscle, macrophages phagocytose necrotic tissue and secrete growth factors including FGF, IGF, HGF, and IL-6 for muscle progenitor cells [27]. In particular, IL-6 is involved in the degradation and degeneration of protein and proliferation of SCs [23]. In this study, IL-6 mRNA expression at 4 h and 3 d was higher in untrained horses than in trained horses, and expression of mRNAs, including HGF, HIF-1*α*, FGF-2, IGF-1, and KDR, was high at 3 d. In previous study in rats [28], hypertrophied plantaris muscle by compensatory overload was associated with increased HGF and FGF-2 mRNA, suggesting that both factors may have an important role in hypertrophy due to SC activation. In another study of human vastus lateralis muscle [29], increased IGF-1 mRNA was found at 3 days after contraction-induced muscle damage, and SC and muscle fiber expressed IGF-1 protein were detected at the same time. Thus, upregulated IGF-1 mRNA is regarded as an important factor for SCs regulation. Subjecting skeletal muscle to injury and inflammation promoted local production of NO by induction of inducible NOS [27]. Given that Pax7, HGF, and HIF-1*α* were upregulated, increases of factors in controls at 3 d suggest that the effects of injury or inflammation are involved in NO production. Conversely, at 3 d, mRNA expression, including IGF-1, FGF-2, IL-6, and HIF-1*α*, was significantly decreased in the Hypo Tr group. These lower levels of expression at 3 d are considered to be due to lower levels of injury or inflammation. This would suggest that higher NO production in the Hypo Tr group might be responsible for decreased injury or inflammation.

Hypoxic conditions may contribute only to the activation of SCs, and subsequent proliferation of SCs probably requires growth factors [10]. Thus, it is thought that increases in growth factors and activation of SCs are suppressed during the late phase in the Hypo Tr group, because hypoxic training attenuates the stress of IET due to a mechanism partly involved with production of NO. On the other hand, the expression of myogenin mRNA was increased in Pre, which might have resulted in a longer duration of SC activation. A previous study [30] in rats demonstrated that myogenin mRNA expression was upregulated until 7 days after muscle damage, and an increase in the number of myogenin-positive nuclei was also found at 3–7 days after the damage. It is not clear which is more essential for angiogenesis, duration or peak SC activation. A previous study [31] that investigated effects of VEGF and IGF-1 delivery on the regenerative process of ischemic muscle demonstrated that sustained delivery (versus a single bolus) has a large impact on muscle repair, including angiogenesis. Therefore, if SCs were activated for a longer period by hypoxic training compared to normoxic training, hypoxic training might contribute to greater angiogenesis. This effect of hypoxic training is probably involved in NO and HIF-1*α*, which activates and inhibits differentiation of SCs. In agreement with increased myogenin mRNA expression in Pre, which is expressed while proangiogenic effects of differentiating SCs are most intense, expression of VEGF-A, ANGPT1, FGF-2, and HGF (which are known to be produced by SCs) increased.

## 5. Conclusion

Hypoxic training produced significant improvements in running distance and ${\dot{V}O}_{2}max$ in the IET under normoxia in horses. Simultaneously, capillary density and mRNA expression including myogenin, VEGF-A, and HGF increased under hypoxic training. These changes induced by training and different oxygen conditions might be caused by NO production and HIF-1*α* expression. We conclude that one of the advantages of hypoxic training is the prolongation of the period during which SCs contribute to angiogenesis.

## Figures and Tables

**Table 1:** Real-time reverse transcriptional-PCR (RT-PCR) primer sequences.

	Forward sequence	Reverse sequence

GAPDH	CAAGGCTGTGGGCAAGGT	GGAAGGCCATGCCAGTGA
Pax7	CATCGGCGGCAGCAA	TCCTCGATCTTTTTCTCCACATC
MyoD	ACGGCTCTCTCTGCAACTTTG	GAGTCGAAACACGGGTCATCA
Myogenin	TCACGGCTGACCCTACAGATG	GGTGATGCTGTCCACAATGG
HIF-1*α*	AAGTGCGAGCACGATTACAGTAT	GACGGTAGGAAGAGCAGGTTCTT
VEGF-A	CCCACTGCGGAGTTCAACAT	TTGGCTTTGGTGAGGTTTGAT
KDR	TTTTGGCATCACGGAAGTGTAT	TTCTCCGACAAGAGGATATTTCG
PGC-1*α*	TCCGTGTCACCACCCAAAT	TGAACGAGAGCGCATCCTT
ANGPT1	GCAAATGTGCCCTCATGCT	CAGATTGGATGGGCCACAAG
HGF	GGTACGCTACGAAGTCTGTGACA	CCCATTGCAGGTCATGCAT
FGF-2	TGCGAACCGTTATCTTGCTATG	CACTCGTCCGTAACACATTTAGAAG
IGF-1	TGTCCTCCTCACATCTCTTCTACCT	CGTGGCAGAGCTGGTGAA
IL-6	AACAACTCACCTCATCCTTCGAA	CGAACAGCTCTCAGGCTGAAC

GAPDH: glyceraldehyde-3-phosphate dehydrogenase; Pax7: paired box transcription factor-7; MyoD: myogenic determination factor; HIF-1*α*: hypoxia inducible factor-1*α*; VEGF-A: vascular endothelial growth factor-A; KDR: vascular endothelial growth factor receptor-2; PGC-1*α*: peroxisome proliferator-activated receptor *γ* coactivator-1*α*; ANGPT1: angiopoietin-1; HGF: hepatocyte growth factor; FGF-2: fibroblast growth factor-2; IGF-1: insulin-like growth factor-1; IL-6: interleukin-6.
